# A Predictive Model for Guillain-Barré Syndrome Based on Single Learning Algorithms

**DOI:** 10.1155/2017/8424198

**Published:** 2017-04-11

**Authors:** Juana Canul-Reich, Juan Frausto-Solís, José Hernández-Torruco

**Affiliations:** ^1^División Académica de Informática y Sistemas, Universidad Juárez Autónoma de Tabasco, Km. 1 Carretera Cunduacán, Jalpa de Méndez, Col. Esmeralda, CP 86690, Cunduacán, TAB, Mexico; ^2^Instituto Tecnológico de Ciudad Madero, Av. 1o. de Mayo esq. Sor Juana Inés de la Cruz s/n, Col. Los Mangos, 89440 Ciudad Madero, TAMPS, Mexico

## Abstract

*Background*. Guillain-Barré Syndrome (GBS) is a potentially fatal autoimmune neurological disorder. The severity varies among the four main subtypes, named as Acute Inflammatory Demyelinating Polyneuropathy (AIDP), Acute Motor Axonal Neuropathy (AMAN), Acute Motor Sensory Axonal Neuropathy (AMSAN), and Miller-Fisher Syndrome (MF). A proper subtype identification may help to promptly carry out adequate treatment in patients.* Method*. We perform experiments with 15 single classifiers in two scenarios: four subtypes' classification and One versus All (OvA) classification. We used a dataset with the 16 relevant features identified in a previous phase. Performance evaluation is made by 10-fold cross validation (10-FCV). Typical classification performance measures are used. A statistical test is conducted in order to identify the top five classifiers for each case.* Results*. In four GBS subtypes' classification, half of the classifiers investigated in this study obtained an average accuracy above 0.90. In OvA classification, the two subtypes with the largest number of instances resulted in the best classification results.* Conclusions*. This study represents a comprehensive effort on creating a predictive model for Guillain-Barré Syndrome subtypes. Also, the analysis performed in this work provides insight about the best single classifiers for each classification case.

## 1. Introduction

Guillain-Barré Syndrome (GBS) is an autoimmune neurological disorder characterized by a fast evolution; usually it goes from a few days up to four weeks. Complications of GBS vary among subtypes, which can be mainly Acute Inflammatory Demyelinating Polyneuropathy (AIDP), Acute Motor Axonal Neuropathy (AMAN), Acute Motor Sensory Axonal Neuropathy (AMSAN), and Miller-Fisher Syndrome (MF) [1, 2].

Current GBS subtype classification method consists of a clinical inspection by physicians guided by criteria established by specialists. This initial diagnostic is reinforced by neuroconduction tests, which help to differentiate among subtypes [1]. This current method implies performing long, expensive, and annoying tests. Some previous efforts in GBS have been focused to predict outcome at 6 months in the acute phase of GBS using clinical characteristics [3], early recognition of poor prognosis [4], and prediction of respiratory insufficiency [5–7]. No publication to date has been found of studies using machine learning methods for GBS subtypes classification.

In this study, we investigate the predictive power of a reduced set of only 16 features selected out from an original dataset of 365 features. This dataset holds data from 129 Mexican patients and contains the four aforementioned GBS subtypes. We apply 15 representative single classifiers from diverse approaches: decision trees (C4.5), instance-based learners (*k*NN: *k* nearest neighbor), kernel-based (SVM: Support Vector Machines), neural networks (SLP, MLP, and RBF-DDA), and rule induction learners (OneR, JRip), among others.

We performed experiments in three classification scenarios: four GBS subtypes' classification, OvA (One versus All), and OvO (One versus One). For clarity purposes and due to page limitation, in this work we present detailed results of the first two scenarios. Details of OvO scenario will be available per reader request.

This study represents a comprehensive effort on creating a predictive model for GBS subtypes. Also, the analysis performed in this work provides insight about the best single classifiers for each classification case. Further experiments with other algorithms will follow.

This paper is organized as follows. In Section 2, we present a description of the dataset, the metrics used in the study, a brief description of the classifiers, the experimental design, and the tuning procedure of classifiers. In Section 3, we show and discuss the experimental results. Finally, in Section 4, we summarize conclusions of the study and also suggest some future work.

## 2. Materials and Methods

### 2.1. Data

The dataset used in this work comprises 129 cases of patients who received treatment at Instituto Nacional de Neurología y Neurocirugía located in Mexico City. There are 20 AIDP cases, 37 AMAN, 59 AMSAN, and 13 Miller-Fisher cases. Hence, there are four GBS subtypes in this dataset.

In a previous work [8], we identified a set of 16 relevant features out of an original 365-feature dataset. The features are listed in Table 1. Features V22, V29, V30, and V31 are all clinical and the remaining features come from a nerve conduction test. The method used to identify these 16 features is briefly described below.

First, we made a preselection of variables from the original dataset based on diagnostic criteria for GBS established in the literature. After preselection, the dataset was left with 156 variables: 121 variables from the nerve conduction test, 4 variables from the CSF analysis, and 31 clinical variables. We used a novel method consisting of a combination of Quenching Simulated Annealing (QSA) and Partitions Around Medoids (PAM), named QSA-PAM method. We used a clustering technique as this method is useful to study the internal structure of data to disclose the existence of groups of homogeneous data. We know in advance of the existence of four GBS subtypes or classes in our dataset; therefore, we took advantage of this information to identify relevant features that allow building four clusters, each corresponding to a GBS subtype. Purity metric was used to determine the quality of each cluster. The highest purity is reached when clusters contain the largest number of elements of the same type and the fewest number of elements of a different type.

QSA [9] is a version of Simulated Annealing (SA), which is a general purpose randomized metaheuristic that finds good approximations to the optimal solution for large combinatorial problems. QSA was used to select different random feature subsets from the dataset. New datasets created using these feature subsets were used as input to PAM to build four clusters. Finally, purity of clusters was measured. Sixteen features from the original dataset were encountered relevant for identifying GBS subtypes with the highest purity, 0.8992.

### 2.2. Single Classifiers

In this study, we include results from 15 representative single classifiers from diverse approaches: decision trees (C4.5), instance-based learners (*k*NN: *k* nearest neighbor), kernel-based (SVM: Support Vector Machines), neural networks (SLP, MLP, and RBF-DDA), rule induction learners (OneR, JRip), and logistic regression, among others. The complete list is given in Table 2, where the tuning parameters are also shown. A detailed description of these classifiers can be found in [10–18]. Experiments from *k*NN, SVM, and C4.5 were previously published [19, 20]. In this work, we used results from these classifiers to make a comparative analysis among all the 15 classifiers. From each approach, we selected the best classifiers based on their performance. The idea is to initially explore different single classifiers to analyze their performance in GBS subtype classification. From the machine learning perspective, it is always useful to analyze the classification power of different classifiers in diverse tasks.

### 2.3. Performance Measures

We used typical performance measures in machine learning such as AUC (Area under the Curve), average accuracy and balanced accuracy. Average accuracy is used in four GBS subtypes' classification, since it is a more suitable measure for multiclass classification problems. Balanced accuracy is used in OvA classification, because it is a better performance estimate of imbalanced datasets.

### 2.4. Experimental Design

We used the 16-feature subset, described in Section 2.1, for experiments. We added the GBS subtype as class variable. Finally, we created a dataset containing the 129 instances and 17 features. We used 10-fold cross validation (10-FCV) evaluation schemes in all cases. We chose this validation scheme since it is more suitable due to our limited dataset. We performed 30 10-FCV runs, for each method listed in Section 2.2. For each fold we computed average accuracy (balanced accuracy for OvA) and AUC (multiclass AUC for four GBS subtype classification). After the 10-fold, we calculated the average of each measure. Finally, we averaged each of these quantities across the 30 runs. In each 10-FCV run, we set a different seed to ensure different splits of train and test sets across runs, then we had all classifiers use the same seed at the same run. These seeds were generated using Mersenne-Twister pseudo-random number generator [21].

We performed experiments in three classification scenarios: four GBS subtypes' classification, OvA, and OvO. For clarity purposes and length of paper, in this work we present detailed results of the first two scenarios. Details of OvO scenario will be available per reader request.

In the first scenario, the four GBS subtypes were included in the dataset at the same time, that is, AIDP, AMAN, AMSAN, and MF. OvA strategy consists of building *n* binary classifiers. In this particular work, we made four different OvA classifications, as the number of GBS subtypes in the dataset. Hence, we created four new datasets. In each dataset, the instances of one class were marked as the positive cases and the instances of the remaining classes were marked as the negative cases. OvO strategy consists of building *n*(*n* − 1)/2 binary classifiers. In this particular work, we made six different OvO classifications. Therefore, we created six new datasets, as many combinations of pairs of GBS subtypes. We aimed to investigate how well classifiers distinguish each subtype with respect to the other subtypes. Each dataset contained instances of only two GBS subtypes, one class marked as the positive case and all remaining classes as the negative case.

## 3. Parameter Optimization

MLP, SLP, RBF-DDA, and JRip each require a particular parameter optimization, as mentioned in Section 2.2. These parameters were automatically optimized by each method in each one of the 30 runs; therefore the best parameters for each run were used for classification.

## 4. Results

### 4.1. Four GBS Subtypes' Classification

In this section, we show the classification results of the four GBS subtypes. All tables show the average results of each classifier across 30 runs. All figures show the average accuracy of each classifier across 30 runs. In both cases, the standard deviation of each metric is shown.

Table 3 shows the four GBS subtypes' classification. Six classifiers, almost half of all the classifiers, obtained an average accuracy above 0.90. The best classifiers were *k*NN, SVMLap, SVMPoly, SVM SVMGaus, C4.5, and SVMLin. Five of the remaining classifiers obtained an average accuracy around 0.89. Two around 0.88 and OneR showed the worst performance with an average accuracy under 0.80 and overall poor results in all metrics.

**Figure 1:**
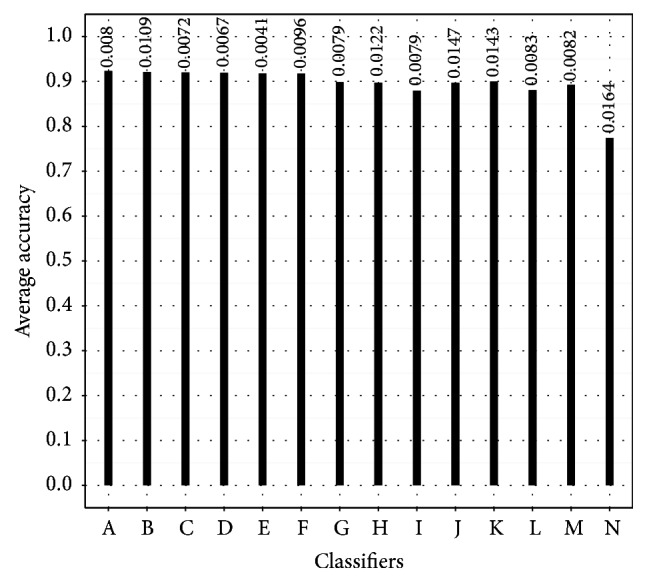
Average accuracy in four GBS subtypes' classification. The standard deviation is shown on top of the bars. A = SVMPoly, B = C4.5, C = SVMLap, D = SVMGaus, E = *k*NN, F = SVMLin, G = Naive Bayes, H = MLP, I = RBF-DDA, J = SLP, K = JRip, L = LDA, M = MLR, and N = OneR.

As Figure 1 show, most of the classifiers obtained an average accuracy around 0.90. Six of them were above this number, and in average, the standard deviation was around 0.01.

### 4.2. OvA Classification Results

In this section, we describe the results of OvA classification. That is, AIDP versus ALL, AMAN versus ALL, AMSAN versus ALL, and MF versus ALL. As mentioned before, we used the balanced accuracy as our base metric in OvA classification scenario. All tables and figures show the average results of each classifier across 30 runs. In all cases, the standard deviation of each metric is shown.

Table 4 shows the average results across 30 runs in OvA classification. In AIDP versus ALL, four classifiers obtained a balanced accuracy above 0.80: *k*NN, C4.5, MLP, and SVMLap. In AMAN versus ALL, nine classifiers obtained a balanced accuracy above 0.90; four of them were SVM with all different kernels. In AMSAN versus ALL, the best five classifiers were *k*NN, C4.5, SVMLap, SLP, and RBF-DDA. They obtained a balanced accuracy above 0.86. MF versus ALL obtained the worst classification performance.

**Figure 2:**
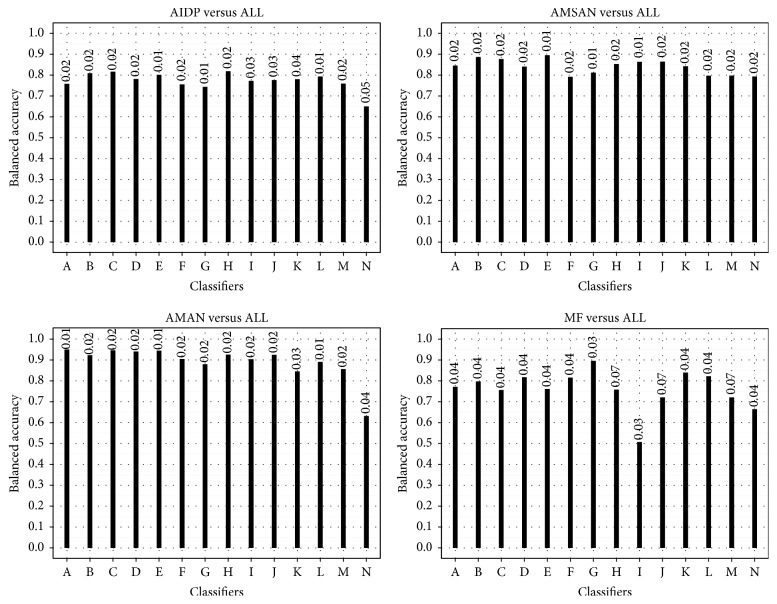
Balanced accuracy in OvA classification. The standard deviation is shown on top of the bars. A = SVMPoly, B = C4.5, C = SVMLap, D = SVMGaus, E = *k*NN, F = SVMLin, G = Naive Bayes, H = MLP, I = RBF-DDA, J = SLP, K = JRip, L = LDA, M = BLR, and N = OneR.

As shown in Figure 2, AMSAN versus ALL showed the most stable performance both in balanced accuracy and in standard deviation across 30 runs. The opposite case was MF versus ALL. AMAN versus ALL obtained the highest classification performance; AMSAN versus ALL was the second best.

### 4.3. Statistical Analysis

We investigated if there was any statistically significant difference in average accuracy among the top five classifiers in average accuracy (balanced accuracy in OvO and OvA scenarios) across 30 runs, in all classification scenarios. For this analysis, we used the Friedman test. An additional post hoc analysis using Holms' correction was performed in cases where null hypothesis was rejected. We selected Friedman test since it is suitable for the type of analysis we performed and also because it is a nonparametric test; that is, no assumption about data distribution is needed. Holm's correction is used for controlling the family-wise error in multiple hypothesis testing. Despite other correction procedures, we selected Holm's because it is a powerful method and it makes no additional assumption about the hypotheses tested. More details about these tests can be found in [22].

Post hoc analysis uses an alpha^*∗*^ parameter, which is the modified alpha value equal to alpha/(*k* − *i*), where alpha is the significance level, *k* is the number of classifiers, and *i* is the rank. In all tests, we used an alpha = 0.05.

#### 4.3.1. Four GBS Subtypes' Classification

In Table 5 we show the Friedman test results of the comparison among the top five classifiers in average accuracy across 30 runs, in four classes classification. The complete list of the top five classifiers for each case is shown in Table 6. No statistically significant difference among the top five classifiers in average accuracy across 30 runs was found.

In all cases, we used as our null hypothesis *H*o: there is no statistically significant difference in the average accuracy among the top five classifiers across 30 runs, and we used as our alternative hypothesis *H*_1_: there is a statistically significant difference in the average accuracy among the top five classifiers across 30 runs.

In Table 6 we show the average ranks for the top five classifiers in four GBS subtypes' classification. As mentioned before, no statistically significant difference among the top five classifiers was found.

In Table 7, the results of the post hoc test with Holm's correction of the top five classifiers in four GBS subtypes' classification are shown. No statistically significant difference between SVMPoly and the other four classifiers was found.

#### 4.3.2. OvA Classification

In Table 8 we show the Friedman test results of the comparison among the top five classifiers in balanced accuracy across 30 runs, in OvA classification. The complete list of the top five classifiers for each case is shown in Table 9. A statistically significant difference among the top five classifiers in balanced accuracy across 30 runs was found in all OvA classifications.

In all cases, we used as our null hypothesis *H*o: there is no statistically significant difference in the balanced accuracy among the top five classifiers across 30 runs, and we used as our alternative hypothesis *H*_1_: there is a statistically significant difference in the balanced accuracy among the top five classifiers across 30 runs.

In Table 9 we show the average ranks for the top five classifiers in OvA classification. We highlight the ranked first classifiers only in cases where a statistically significant difference was found. The ranked first classifiers were MLP (2.17) for AIDP versus ALL, SVMPoly (2.37) for AMAN versus ALL, *k*NN (1.40) for AMSAN versus ALL, and Naive Bayes (1.20) for MF versus ALL.

In Table 10, the results of the post hoc test with Holm's correction of the top five classifiers in AIDP versus ALL classification are shown. A statistically significant difference between MLP and LDA was found, as well as between MLP and *k*NN.

In Table 11, the results of the post hoc test with Holm's correction of the top five classifiers in AMAN versus ALL classification are shown. A statistically significant difference between SVMPoly and MLP was found.

In Table 12, the results of the post hoc test with Holm's correction of the top five classifiers in AMSAN versus ALL classification are shown. A statistically significant difference between *k*NN and the rest of classifiers was found.

In Table 13, the results of the post hoc test with Holm's correction of the top five classifiers in MF versus ALL classification are shown. A statistically significant difference between Naive Bayes and the rest of classifiers was found.

## 5. Discussion

Our objective in this work was to create the highest accurate predictive model for GBS possible, using the 16 relevant features identified with QSA-PAM method. This work constitutes the first effort on this topic using machine learning methods. For this first approach, we used single classifiers. We selected 15 single classifiers from diverse types: decision trees (C4.5), instance-based learners (*k*NN), kernel-based (SVM), neural networks (SLP, MLP, and RBF-DDA), and rule induction learners (OneR, JRip), among others. The complete list is in Section 2.2. We compared their performance in three types of experiments: four GBS subtypes' classification, OvA classification, and OvO classification.

### 5.1. Four GBS Subtypes' Classification

The best classifiers were *k*NN and SVM with all different kernels and C4.5. This result confirms them as a good single classifier. The standard deviation of the average accuracy was low; this could be a consequence of the cross validation characteristic in the sense of reducing the variance by averaging over *k* different partitions.

OneR obtained the worst performance. One possible explanation of this situation is that, since OneR generates one single rule to make the classification, maybe that single rule is not enough to classify the four classes in this particular problem.

After the statistical analysis, no statistical significant difference in average accuracy among the top five classifiers across 30 runs was found. One possible explanation for this last result could be the stability in average accuracy achieved by the classifiers in 10-FCV.

### 5.2. OvA Classification

AMAN versus ALL showed the highest performance in balanced accuracy across 30 runs. The opposite case was MF versus ALL. AMSAN versus ALL was the second best. The two classes with the largest number of instances resulted in the best classification results, that is, AMAN versus ALL and AMSAN versus ALL. *k*NN, C4.5, and SVMLap appear in the top five classifiers in most cases. Naive Bayes appears as the top classifier for MF versus ALL. In most cases, OneR obtained the worst performance. Overall, the highest and more stable average results across 30 runs were obtained in 10-FCV scenario.

After the statistical analysis, two classifiers stood out from the rest. Naive Bayes resulted as the best classifier for the minority class versus ALL, that is, MF versus ALL. *k*NN was the best classifier for AMSAN versus ALL.

## 6. Conclusions

In this work, we aimed at creating the highest accurate predictive model for GBS possible, using the 16 relevant features identified with QSA-PAM method. This work constitutes the first effort on this topic using machine learning methods. Using a reduced set of predictors for GBS subtypes could result in applying simpler and faster medical tests.

For this first approach, we used single classifiers. We selected 15 single classifiers from diverse types: decision trees (C4.5), instance-based learners (*k*NN), kernel-based (SVM), neural networks (SLP, MLP, and RBF-DDA), and rule induction learners (OneR, JRip), among others. The complete list is in Section 2.2. We compared their performance in three types of experiments: four GBS subtypes' classification, OvA classification, and OvO classification. However, in this work we only present results from the two first scenarios.

In four GBS subtypes' classification, we obtained an average accuracy ≥ 0.90 with half of classifiers investigated. In OvA classification, the two classes with the largest number of instances resulted in the best classification results, that is, AMAN versus ALL and AMSAN versus ALL. Although some classifiers stand out from the rest, as mentioned in Discussion, each classification scenario obtained a best classification method. The analysis performed in this work provides insight about the best classifiers for each classification case. Furthermore, from the machine learning perspective, it is always useful to analyze the classification power of different classifiers in diverse tasks.

This study is limited with regard to the quantity of instances present in the dataset. Another limitation is the absence of other GBS datasets to compare with our results.

As future work, we will investigate the performance of ensemble methods. Also, we will further tackle the imbalanced data problem.

## Figures and Tables

**Table 1:** List of features used in this study.

Feature label	Feature name
v22	Symmetry (in weakness)
v29	Extraocular muscles involvement
v30	Ptosis
v31	Cerebellar involvement
v63	Amplitude of left median motor nerve
v106	Area under the curve of left ulnar motor nerve
v120	Area under the curve of right ulnar motor nerve
v130	Amplitude of left tibial motor nerve
v141	Amplitude of right tibial motor nerve
v161	Area under the curve of right peroneal motor nerve
v172	Amplitude of left median sensory nerve
v177	Amplitude of right median sensory nerve
v178	Area under the curve of right median sensory nerve
v186	Latency of right ulnar sensory nerve
v187	Amplitude of right ulnar sensory nerve
v198	Area under the curve of right sural sensory nerve

**Table 2:** List of single classifiers used in this study. Binary Logistic Regression (BLR) used in OvA and OvO classifications. Multinomial Logistic Regression (MLR) used in four GBS subtypes' classification.

Single classifier	Approach	Tuning parameter
*k*NN	Instance-based	*k*, *d*
SVM Linear kernel (SVMLin)	Kernel-based	*C*
SVM Polynomial kernel (SVMPoly)	Kernel-based	*C*, degree, *σ* (*γ*), coef
SVM Gaussian kernel (SVMGaus)	Kernel-based	*C*, *σ* (*γ*)
SVM Laplacian kernel (SVMLap)	Kernel-based	*C*, *σ* (*γ*)
C4.5	Decision tree	NA
Single Layer Perceptron (SLP)	Neural network	Size, decay
Multilayer Perceptron (MLP)	Neural network	Size
Radial Basis Function ANN (RBF-ANN)	Neural network	Negative threshold
JRip	Rule induction	NumOpt
OneR	Rule induction	NA
Naive Bayes	Bayesian	NA
Binary Logistic Regression (BLR)	Regression	NA
Multinomial Logistic Regression (MLR)	Regression	NA
Linear Discriminant Analysis (LDA)	Discriminant Analysis	NA

**Table 3:** Four GBS subtypes' classification. The standard deviation of each metric is shown in normal font.

Classifier	Optimal parameters	Average accuracy	Multiclass AUC
SVMPoly	*d* = 6 coef = 1	**0.9235**	**0.8985**
	*g* = 0.01 *C* = 1	0.0080	0.0199
C4.5		**0.9211**	**0.8857**
		0.0109	0.0242
SVMLap	*s* = 0.01 *C* = 50	**0.9201**	**0.8712**
		0.0072	0.0240
SVMGaus	*s* = 0.01 *C* = 10	**0.9193**	**0.8897**
		0.0067	0.0221
*k*NN	*k* = 14 *d* = 1	**0.9179**	**0.8783**
		0.0041	0.0188
SVMLin	*C* = 1	**0.9175**	**0.8632**
		0.0096	0.0232
JRip		**0.8999**	**0.8729**
		0.0143	0.0291
Naive Bayes		**0.8986**	**0.8632**
		0.0079	0.0244
MLP		**0.8974**	**0.8514**
		0.0122	0.0257
SLP		**0.8972**	**0.8452**
		0.0147	0.0230
MLR		**0.8926**	**0.8405**
		0.0082	0.0279
LDA		**0.8806**	**0.8256**
		0.0083	0.0223
RBF-DDA		**0.8797**	**0.8249**
		0.0079	0.0287
OneR		**0.7744**	**0.7528**
		0.0164	0.0249

**Table 4:** OvA classification results. The standard deviation of each metric is shown in normal font.

AMAN versus ALL			AMSAN versus ALL			AIDP versus ALL			MF versus ALL		
Classifier	Balanced accuracy	AUC	Classifier	Balanced accuracy	AUC	Classifier	Balanced accuracy	AUC	Classifier	Balanced accuracy	AUC
SVMPoly	**0.9498**	**0.9498**	*k*NN	**0.8951**	**0.8951**	MLP	**0.8183**	**0.8183**	Naive Bayes	**0.8956**	**0.8956**
	0.0135	0.0135		0.0124	0.0124		0.0204	0.0204		0.0252	0.0252
SVMLap	**0.9459**	**0.9459**	C4.5	**0.8860**	**0.8860**	SVMLap	**0.8158**	**0.8158**	JRip	**0.8395**	**0.8395**
	0.0173	0.0173		0.0163	0.0163		0.0214	0.0214		0.0424	0.0424
*k*NN	**0.9441**	**0.9441**	SVMLap	**0.8767**	**0.8767**	C4.5	**0.8083**	**0.8083**	LDA	**0.8218**	**0.8218**
	0.0067	0.0067		0.0189	0.0189		0.0226	0.0226		0.0371	0.0371
SVMGaus	**0.9400**	**0.9400**	SLP	**0.8647**	**0.8647**	*k*NN	**0.8012**	**0.8012**	SVMGaus	**0.8168**	**0.8168**
	0.0177	0.0177		0.0229	0.0229		0.0135	0.0135		0.0397	0.0397
MLP	**0.9256**	**0.9256**	RBF-DDA	**0.8629**	**0.8629**	LDA	**0.7928**	**0.7928**	SVMLin	**0.8150**	**0.8150**
	0.0180	0.0180		0.0138	0.0138		0.0138	0.0138		0.0438	0.0438
SLP	**0.9244**	**0.9244**	MLP	**0.8527**	**0.8527**	SVMGaus	**0.7807**	**0.7807**	C4.5	**0.7971**	**0.7971**
	0.0193	0.0193		0.0180	0.0180		0.0222	0.0222		0.0446	0.0446
C4.5	**0.9224**	**0.9224**	SVMPoly	**0.8454**	**0.8454**	JRip	**0.7800**	**0.7800**	SVMPoly	**0.7711**	**0.7711**
	0.0199	0.0199		0.0183	0.0183		0.0403	0.0403		0.0420	0.0420
SVMLin	**0.9046**	**0.9046**	JRip	**0.8420**	**0.8420**	SLP	**0.7753**	**0.7753**	*k*NN	**0.7609**	**0.7609**
	0.0244	0.0244		0.0212	0.0212		0.0323	0.0323		0.0426	0.0426
RBF-DDA	**0.9033**	**0.9033**	SVMGaus	**0.8403**	**0.8403**	RBF-DDA	**0.7715**	**0.7715**	MLP	**0.7579**	**0.7579**
	0.0194	0.0194		0.0184	0.0184		0.0254	0.0254		0.0695	0.0695
LDA	**0.8902**	**0.8902**	Naive Bayes	**0.8112**	**0.8112**	BLR	**0.7588**	**0.7588**	SVMLap	**0.7556**	**0.7556**
	0.0125	0.0125		0.0140	0.0140		0.0233	0.0233		0.0422	0.0422
Naive Bayes	**0.8794**	**0.8794**	BLR	**0.7969**	**0.7969**	SVMPoly	**0.7578**	**0.7578**	SLP	**0.7211**	**0.7211**
	0.0182	0.0182		0.0188	0.0188		0.0228	0.0228		0.0659	0.0659
BLR	**0.8556**	**0.8556**	LDA	**0.7963**	**0.7963**	SVMLin	**0.7552**	**0.7552**	BLR	**0.7211**	**0.7211**
	0.0197	0.0197		0.0152	0.0152		0.0204	0.0204		0.0659	0.0659
JRip	**0.8454**	**0.8454**	OneR	**0.7925**	**0.7925**	Naive Bayes	**0.7432**	**0.7465**	OneR	**0.6641**	**0.6641**
	0.0312	0.0312		0.0191	0.0191		0.0100	0.0155		0.0403	0.0403
OneR	**0.6313**	**0.6339**	SVMLin	**0.7916**	**0.7922**	OneR	**0.6497**	**0.6517**	RBF-DDA	**0.5071**	**0.5071**
	0.0404	0.0413		0.0192	0.0195		0.0486	0.0489		0.0288	0.0288

**Table 5:** Friedman test results of the comparison among top five classifiers in average accuracy across 30 runs in four GBS subtypes' classification.

Friedman Statistic	Critical value	*H*o
1.985	2.45	Accepted

**Table 6:** Average ranks of the top five classifiers in four GBS subtypes' classification.

	Classifiers compared				
	SVMPoly	C4.5	SVMLap	SVMGauss	*k*NN
Average ranks	2.47	2.87	2.87	3.38	3.42

**Table 7:** Post hoc test with Holm's correction of the top five classifiers in four GBS subtype classification.

Classifiers compared	*p* value
SVMPoly versus *k*NN	0.020
SVMPoly versus SVMGauss	0.025
SVMPoly versus C4.5	0.327
SVMPoly versus SVMLap	0.327

**Table 8:** Friedman test results of the comparison among top five classifiers in balanced accuracy across 30 runs in OvA classification.

Classes	Friedman Statistic	Critical value
AIDP versus ALL	8.989	2.45
AMAN versus ALL	8.651	2.45
AMSAN versus ALL	35.869	2.45
MF versus ALL	25.591	2.45

**Table 9:** Average ranks of the top five classifiers in OvA classification.

Classes	Classifiers compared				
AIDP versus ALL	MLP	SVMLap	C4.5	*k*NN	LDA
*Average ranks *	2.17	2.38	2.92	3.53	4.00
AMAN versus ALL	SVMPoly	SVMLap	*k*NN	SVMGaus	MLP
*Average ranks *	2.37	2.55	2.78	3.02	4.28
AMSAN versus ALL	*k*NN	C4.5	SVMLap	SLP	RBF-DDA
*Average ranks *	1.40	2.23	3.22	3.97	4.18
MF versus ALL	Naive Bayes	JRip	LDA	SVMGaus	SVMLin
*Average ranks *	1.20	2.92	3.78	3.22	3.88

**Table 10:** Post hoc test with Holm's correction of the top five classifiers in AIDP versus ALL classification.

Classes	Classifiers compared	*p* value
AIDP versus ALL	MLP versus LDA	0.000
	MLP versus *k*NN	0.001
	MLP versus C4.5	0.066
	MLP versus SVMLap	0.596

**Table 11:** Post hoc test with Holm's correction of the top five classifiers in AMAN versus ALL classification.

Classes	Classifiers compared	*p* value
AMAN versus ALL	SVMPoly versus MLP	0.000
	SVMPoly versus SVMGaus	0.111
	SVMPoly versus *k*NN	0.307
	SVMPoly versus SVMLap	0.653

**Table 12:** Post hoc test with Holm's correction of the top five classifiers in AMSAN versus ALL classification.

Classes	Classifiers compared	*p* value
AMSAN versus ALL	*k*NN versus RBF-DDA	0.000
	*k*NN versus SLP	0.000
	*k*NN versus SVMLap	0.000
	*k*NN versus C4.5	0.041

**Table 13:** Post hoc test with Holm's correction of the top five classifiers in MF versus ALL classification.

Classes	Classifiers compared	*p* value
MF versus ALL	Naive Bayes versus SVMLin	0.000
	Naive Bayes versus LDA	0.000
	Naive Bayes versus SVMGaus	0.000
	Naive Bayes versus JRip	0.000
